# Rationale and methods of a randomized trial evaluating the effect of neprilysin inhibition on left ventricular remodelling

**DOI:** 10.1002/ehf2.13137

**Published:** 2020-12-10

**Authors:** Kieran F. Docherty, Ross T. Campbell, Katriona J.M. Brooksbank, Rosemary L. Godeseth, Paul Forsyth, Alex McConnachie, Giles Roditi, Bethany Stanley, Paul Welsh, Pardeep S. Jhund, Mark C. Petrie, John J.V. McMurray

**Affiliations:** ^1^ Institute of Cardiovascular and Medical Sciences, BHF Glasgow Cardiovascular Research Centre University of Glasgow Glasgow G12 8TA UK; ^2^ Queen Elizabeth University Hospital Glasgow UK; ^3^ Pharmacy Services NHS Greater Glasgow and Clyde Glasgow UK; ^4^ Robertson Centre for Biostatistics University of Glasgow Glasgow UK; ^5^ Department of Radiology Glasgow Royal Infirmary Glasgow UK

**Keywords:** Clinical trial, Heart failure, Myocardial infarction, Natriuretic peptides, Neprilysin, Renin angiotensin aldosterone system

## Abstract

**Aims:**

In patients at high risk of heart failure following myocardial infarction (MI) as a result of residual left ventricular systolic dysfunction (LVSD), the angiotensin receptor neprilysin inhibitor sacubitril/valsartan may result in a greater attenuation of adverse left ventricular (LV) remodelling than renin angiotensin aldosterone system inhibition alone, due to increased levels of substrates for neprilysin with vasodilatory, anti‐hypertrophic, anti‐fibrotic, and sympatholytic effects.

**Methods:**

We designed a randomized, double‐blinded, active‐comparator trial to examine the effect of sacubitril/valsartan to the current standard of care in reducing adverse LV remodelling in patients with asymptomatic LVSD following MI. Eligible patients were ≥3 months following MI, had an LV ejection fraction ≤40% as measured by echocardiography, were New York Heart Association functional classification I, tolerant of an angiotensin‐converting enzyme inhibitor or angiotensin receptor blocker at equivalent dose of ramipril 2.5 mg twice daily or greater, and taking a beta‐blocker unless contraindicated or intolerant. Patients were randomized to sacubitril/valsartan (target dose 97/103 mg twice daily) or valsartan (target dose 160 mg twice daily). The primary endpoint will be change in LV end‐systolic volume indexed for body surface area measured using cardiac magnetic resonance imaging over 52 weeks from randomization. Secondary endpoints include other magnetic resonance imaging‐based metrics of LV remodelling, biomarkers associated with LV remodelling and neurohumoral activation, and change in patient well‐being assessed using a patient global assessment questionnaire.

**Conclusions:**

This trial will investigate the effect of neprilysin inhibition on LV remodelling and the neurohumoral actions of sacubitril/valsartan in patients with asymptomatic LVSD following MI.

## Introduction

Routine use of coronary reperfusion therapy (initially with thrombolysis and latterly with percutaneous intervention) has reduced the degree of ventricular damage sustained at the time of acute myocardial infarction (MI) and improved survival.^1^ Despite this, the development of left ventricular systolic dysfunction (LVSD) and subsequent heart failure (HF) after acute infarction remains relatively common.^2^, ^3^ The key mechanism underlying development of HF with reduced ejection fraction (HFrEF) following MI is the process of pathological left ventricular (LV) remodelling.^4^ It is generally accepted that patients destined to develop HFrEF after MI experience progressive LV enlargement and reduction in LV ejection fraction (LVEF), developing over weeks, months, years, or even decades after their acute coronary event. It is also accepted that such patients may remain symptomless for a long period, despite significant LV enlargement and LVSD. The prevention of adverse remodelling through pharmacological inhibition of the maladaptive neurohumoral system activation in patients at high risk of HFrEF following an MI has been demonstrated to reduce the risk of developing HFrEF and death.^5^ Indeed, four different neurohumoral antagonists [angiotensin‐converting enzyme (ACE) inhibitors or angiotensin receptor blockers (ARBs), beta‐blockers, and mineralocorticoid receptor antagonists] are life‐saving in both patients at high risk of HF following MI and with established chronic HFrEF.^5^, ^6^, ^7^, ^8^, ^9^, ^10^, ^11^, ^12^, ^13^, ^14^, ^15^, ^16^, ^17^, ^18^ This is unsurprising given that, in many patients, HFrEF is part of the same physiological continuum initiated at the time of acute MI.

Not all neurohumoral systems activated in patients after MI (or in HF) are harmful, and some endogenous neurohumoral systems may be protective. A‐type natriuretic peptide (ANP) and B‐type natriuretic peptide (BNP) are secreted by the heart in response to increased wall stress, and these peptides promote vasodilation (reducing LV wall stress), stimulate renal sodium and water excretion (i.e. antagonizing the retention of salt and water characterizing HF), and inhibit pathological growth, that is, hypertrophy and fibrosis (key components of the adverse LV remodelling that occurs after MI and in HFrEF).^19^ The augmentation of plasma levels of endogenous natriuretic peptides can be achieved through inhibition of neprilysin, the enzyme responsible for the breakdown of natriuretic peptides. In the Prospective Comparison of ARNI (Angiotensin Receptor Neprilysin Inhibitor) with ACE inhibitor to Determine Impact on Global Mortality and Morbidity in Heart Failure Trial (PARADIGM‐HF), the addition of neprilysin inhibition to blockade of the renin angiotensin aldosterone system (RAAS) (using sacubitril/valsartan), compared with RAAS blockade alone (using the gold‐standard ACE inhibitor enalapril), reduced the risk of HF hospitalization and cardiovascular death in patients with HFrEF.^20^ Given their vasodilatory, anti‐hypertrophy, anti‐fibrotic, and sympatholytic effects, along with the clinical benefits observed in patients with HFrEF, the augmentation of natriuretic peptides and other substrates for neprilysin (adrenomedullin, apelin, and glucagon‐like peptide‐1, among others) with a neprilysin inhibitor presents an attractive therapeutic proposal in patients with asymptomatic LVSD after MI, in the hope of delaying or preventing progression to HFrEF. Key to such a therapeutico benefit would be attenuation of LV remodelling over time.

To further explore this hypothesis, we have designed a randomized‐controlled trial comparing the angiotensin receptor neprilysin inhibitor, sacubitril/valsartan, with the ARB, valsartan, to provide information on the effect of neprilysin inhibition on LV remodelling in patients at high risk after MI as a result of residual LVSD.

## Methods

### Trial organization and sources of funding

The trial was conceived and designed by the Trial Steering Committee. The trial was co‐sponsored by the University of Glasgow and the National Health Service Greater Glasgow and Clyde health board. The trial protocol and any substantial amendments to the protocol were approved by the East of Scotland Research Ethics Committee.

This trial was funded by the British Heart Foundation (PG/17/23/32850), and trial medication along with funding for trial drug packaging, labelling, distribution, storage and destruction was supplied by Novartis Pharmaceuticals UK limited who had no input to the design. J. J. V. M. and M. C. P. are supported by a British Heart Foundation Centre of Research Excellence Grant (RE/18/6/34217).

### Trial design

The trial is a multi‐centre, prospective, randomized, double‐blind, active‐comparator trial designed to evaluate the effect of sacubitril/valsartan at a target dose of 97/103 mg twice daily, compared with valsartan at a target dose of 160 mg twice daily, on LV remodelling in patients with asymptomatic LVSD following MI, considered to be at high risk of developing HFrEF. The trial is registered as ClinicalTrials.gov Identifier: NCT03552575.

### Patient eligibility and consent

Consenting patients at least 3 months after acute MI (defined according to the third universal definition of MI^21^) was eligible if they had an LVEF ≤40% as measured by transthoracic echocardiography without any symptoms of HF (i.e. New York Heart Association functional classification I), were taking a minimum dose of ACE inhibitor/ARB (ramipril 2.5 mg BD or equivalent, Supporting Information, *Table S1*), or were able to tolerate such a dose, were treated with a beta‐blocker, unless intolerant or contraindicated, and had a systolic blood pressure ≥100 mmHg. Patients were ineligible if they had permanent or persistent atrial fibrillation, an estimated glomerular filtration rate of <30 mL/min/1.73m^2^, and/or a serum potassium level of >5.2 mmol/L. Full inclusion and exclusion criteria are detailed in *Table* *1*.

**Table 1:** Trial inclusion and exclusion criteria

Inclusion criteria	● Acute myocardial infarction ≥3 months prior to randomization ● Left ventricular ejection ≤40% as measured by transthoracic echocardiography ● Ability to provide written, informed consent ● Age ≥18 years ● Tolerance of a minimum dose of ACE inhibitor/ARB (ramipril 2.5 mg twice daily or equivalent) ● Treatment with a beta‐blocker unless not tolerated or contraindicated
Exclusion criteria	● Contraindication to cardiac MRI (ferrous prosthesis, implantable cardiac device, or severe claustrophobia) ● Clinical (NYHA functional class ≥II) and/or radiological heart failure ● Symptomatic hypotension and/or systolic blood pressure <100 mmHg ● eGFR <30 mL/min/1.73m^2^ and/or serum potassium >5.2 mmol/L ● Persistent/permanent atrial fibrillation ● History of acute myocardial infarction within last 3 months ● History of hypersensitivity or allergy to ACE inhibitors/ARB ● History of angioedema ● Known hypersensitivity to the active study drug substances, contrast media, or any of the excipients ● Obesity (where body girth exceeds MRI scanner diameter) ● Pregnancy, planning pregnancy, or breast feeding ● Inability to give informed consent or comply with study protocol ● Evidence of hepatic disease as determined by any one of the following: AST or ALT values exceeding 2 × ULN at Visit 1, history of hepatic encephalopathy, history of oesophageal varices, or history of portacaval shunt ● History of biliary cirrhosis and cholestasis ● Active treatment with cholestyramine or colestipol resins ● Active treatment with lithium or direct renin inhibitor ● Participation in another intervention study involving a drug or device within the past 90 days (co‐enrolment in observational studies is permitted)

ACE, angiotensin‐converting enzyme; ALT, alanine aminotransferase; ARB, angiotensin receptor blocker; AST, aspartate aminotransferase; eGFR, estimated glomerular filtration rate; MRI, magnetic resonance imaging; NYHA, New York Heart Association; ULN, upper limit of normal.

#### Echocardiography

Non‐contrast transthoracic echocardiography was performed in the left lateral decubitus position, and LVEF measured using Simpson's biplane method.^22^ Patients with insufficient endocardial definition to allow accurate planimetry were excluded.

### Trial procedures

**Figure 1:**
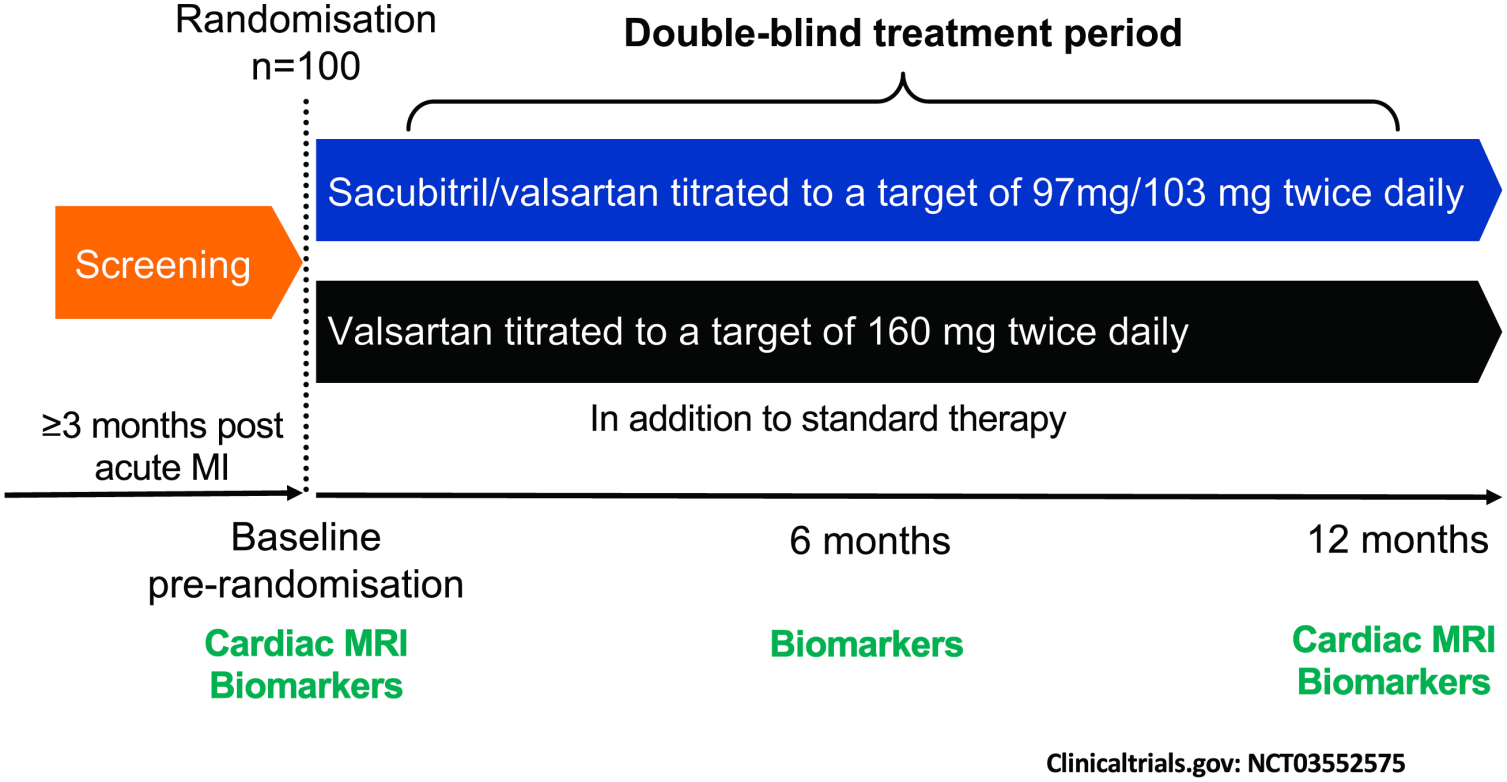
Trial outline. Outline of the trial procedures. Eligible patients were randomized to either sacubitril/valsartan (target dose 97/103 mg twice daily) or valsartan (target dose 160 mg twice daily) and matched placebo for 12 months. Cardiac magnetic resonance imaging (MRI) was performed pre‐randomization and at 12 months. Blood and urine collection for biomarker profiling was performed pre‐randomization, at 6 and 12 months. MI, myocardial infarction.

Prior to randomization, patients had a series of baseline investigations including measurement of height, weight, a physical examination, 12‐lead electrocardiograph, spot urine sample collection and blood sampling for clinical biochemistry, full blood count, and biomarker analysis. Following randomization, study visits took place at 1, 2, 4, 5, 14, 26, 39, and 52 weeks as detailed in *Figure* *1* and Supporting Information, *Table* *S2*, with a particular focus on safety measurements (blood pressure, potassium, and renal function measurements) and the occurrence of any adverse events.

#### Cardiac magnetic resonance imaging

**Figure 2:**
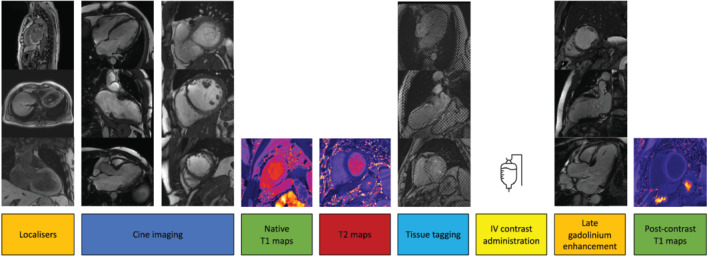
Cardiac magnetic resonance imaging protocol outline. Cardiac magnetic resonance imaging was performed pre‐randomization and at 12 months following double‐blind treatment with sacubitril/valsartan or valsartan. All scans were performed on a single, 3 Tesla Siemens MAGNETOM Prisma scanner. Further details regarding the scan protocol are available in the Supporting Information.

Cardiac magnetic resonance imaging (MRI) was performed prior to randomization and at 12 months following randomization with a single 3 Tesla Siemens MAGNETOM Prisma scanner at the Queen Elizabeth University Hospital Glasgow Imaging Centre of Excellence. Images were obtained with a phased‐array chest coil, during breath‐hold, and gated to the electrocardiogram. The MRI protocol is outlined in *Figure* *2*, and detailed information is provided in the Supporting Information. A single operator (R. T. C.), accredited by the European Association of Cardiovascular Imaging in cardiac MRI analysis with >5 years of experience, analysed all scans blinded to treatment assignment. The baseline and 12 months of follow‐up scans were analysed in pairs to reduce intra‐observer variability, using the methods detailed in the Supporting Information and in accordance with the Society for Cardiovascular Magnetic Resonance and European Society of Cardiovascular Imaging guidelines for reporting cardiovascular magnetic resonance examinations.^23^, ^24^ A random selection of scans (10%) was analysed by a second operator blinded to treatment assignment for assessment of inter‐operator variability and quality assurance. All scans were reviewed by a third operator (G. R.) for the purposes of a clinical report, review of non‐cardiac elements, and the presence of any incidental findings.

#### Biomarkers

Venous blood and spot urine samples were collected at baseline and at 6 and 12 months following randomization (*Figure* *1*). Samples were collected in chilled tubes and centrifuged immediately at 1500 g at 4°C for 10 min before aliquoting and storage at −80°C. A protease inhibitor (Aprotinin, Abcam, Cambridge, UK) was added to ethylenediaminetetraacetic acid plasma to minimize degradation of labile peptides such as ANP.

#### Patient global assessment of change

Patients completed a patient global assessment of change questionnaire at the 12 months of visit. Details of the questionnaire and available responses are in the Supporting Information.

#### Randomization and blinding

Following baseline measurements, participants were randomly assigned to sacubitril/valsartan or valsartan in a 1:1 ratio. Patients were provided with two packs of tablets—valsartan or matching placebo and sacubitril/valsartan or matching placebo—and instructed to take one pill from each pack (i.e. one active treatment and one placebo pill) twice daily. Randomization was stratified by baseline left ventricular end‐systolic volume index (LVESVI) measured using cardiac MRI (≤45 mL/m^2^/>45 mL/m^2^) and by use of diuretics. The randomization schedule was generated by a computer using permuted blocks, with block lengths of 4 and 6. All participants and trial staff were blind to treatment allocation.

#### Patients taking an angiotensin‐converting enzyme inhibitor at baseline

In order to minimize the risk of angioedema due to overlapping ACE and neprilysin inhibition, all patients taking an ACE inhibitor at baseline underwent a 36 h ‘washout’ period following randomization, prior to the first dose of study drug. For the same reason, use of open‐label ACE inhibitor or ARB in addition to the randomized study drug was strictly prohibited for the duration of the trial.

#### Dose adjustment

**Figure 3:**
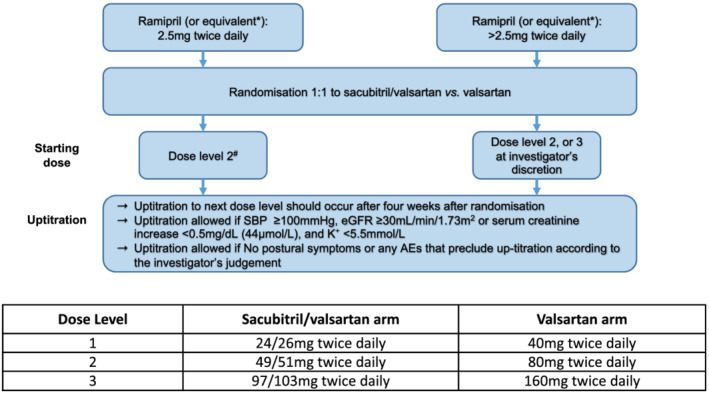
Trial drug initiation and up‐titration. *Equivalent doses detailed in Supporting Information, *Table S1*. ^#^Dose Level 1 could be considered for patients with systolic blood pressure (SBP) ≥100 to 110 mmHg and/or moderate renal impairment [estimated glomerular filtration rate (eGFR) of 30–60 mL/min/1.73 m^2^] at time of randomization. AEs, adverse events.

Three dose levels of study medication were available, with planned stepwise up‐titration (*Figure* *3*). Study drug was started at Dose Level 2 (sacubitril/valsartan 49 mg/51 mg twice daily or valsartan 80 mg twice daily) and up‐titrated after 4 weeks to Dose Level 3 (sacubitril/valsartan 97 mg/103 mg twice daily or valsartan 160 mg twice daily) if tolerated as assessed by clinical review (systolic blood pressure and symptomatic hypotension) and laboratory evaluation (potassium and renal function). Patients already on a high dose of ACE inhibitor/ARB could start at Dose Level 3 at the investigator's discretion.

Alternatively, patients could be started at Dose Level 1 (sacubitril/valsartan 24 mg/26 mg twice daily), with a two‐step titration to target dose over Visits 3 and 4, if systolic blood pressure at Visit 1 was 100 to 110 mmHg or if estimated glomerular filtration rate was 30–60 mL/min/1.73 m^2^. Down‐titration was possible during follow‐up, but the goal was to maintain patients on Dose Level 3 for as much of the trial as possible. Initial up‐titration was only halted (or dose subsequently decreased) because of safety or tolerability concerns related to (i) symptomatic hypotension, (ii) a clinically significant decline in renal function, or (iii) hyperkalaemia (*Figure* *3*).

Patients continued standard background therapy.

#### Development of heart failure and study drug/study discontinuation during follow‐up

Patients developing HF during follow‐up were offered open‐label sacubitril/valsartan. Patients starting open‐label sacubitril/valsartan or withdrawing from study medication (or study follow‐up) ≥6 months after randomization were asked to undergo an ‘end‐of‐study’ cardiac MRI examination (patients withdrawing before 6 months were not asked to have a second cardiac MRI as an effect of LV remodelling was unlikely to be detected before that time point).

### Trial endpoints

The primary and secondary endpoints will be measured as change from baseline to 12 months of follow‐up. The between‐treatment differences in these changes will be analysed.

#### Primary endpoint

The primary endpoint is change in LV end‐systolic volume, measured using cardiac MRI, and indexed for body surface area (LVESVI).

#### Secondary endpoints

The pre‐specified secondary endpoints are as follows:

- change in N‐terminal pro‐B‐type natriuretic peptide;
- change in high‐sensitivity troponin I;
- change in other cardiac MRI‐based metrics of LV remodelling:
  - LV end‐diastolic volume indexed for body surface area
  - left atrial volume indexed for body surface area
  - LVEF
  - LV mass index; and
- change in patient well‐being, assessed using a patient global assessment questionnaire.

#### Exploratory endpoints

The exploratory endpoints are as follows:

- change in biomarkers of LV remodelling: soluble suppression of tumourigenicity‐2 (ST2), galectin‐3, tissue inhibitor matrix metalloproteinase‐1 (TIMP‐1), matrix metallopeptidase‐9 (MMP‐9), Type III procollagen peptide, growth differentiation factor‐15 (GDF‐15), and other relevant biomarkers of interest;
- change in neurohormonal levels: BNP, mid‐regional pro‐ANP, C‐terminal ANP, C‐type natriuretic peptide, mid‐regional pro‐adrenomedullin, guanosine 3',5' cyclic monophosphate (cGMP), endothelin‐1, neprilysin antigen, renin and aldosterone, and other relevant biomarkers of interest; and
- change in extracellular volume fraction, LV global function index, T1 relaxation time, and LV strain as measured using cardiac MRI.

### Statistical considerations

The study size was 100 patients, based on the calculation that 45 patients in each treatment group provided >90% power (*α* level = 0.05) to detect a difference of 6 mL/m^2^ in LVESVI (standard deviation = 7.8 mL/m^2^),^25^ accounting for a discontinuation rate of 10% (lost to follow‐up, development of HF, or death). A 6 mL/m^2^ difference in LVESVI was selected as it is believed to represent a minimally important difference.^26^, ^27^

The primary analysis will include all patients randomized with baseline and 12 months of outcome data on an intention‐to‐treat basis with no imputation for missing data. Each efficacy outcome will be analysed using a regression analysis model adjusted for the baseline value of the outcome in question (e.g. LVESVI) and use of diuretic at baseline. MRI outcomes will also include adjustment for the time from baseline to follow‐up MRI. Efficacy outcome measures will be summarized at baseline, 12 months, and for the change from baseline to 12 months, presented overall and by treatment group. The effect of treatment will be presented with 95% confidence intervals and a two‐sided *P*‐value.

## Discussion

Results from this trial will provide detailed insight into the effects of neprilysin inhibition, added to standard care, in patients at high risk of developing HF as a result of residual LVSD following MI. It will be the first adequately powered, randomized, and long‐term examination of the effect of sacubitril/valsartan on LV remodelling and the only comparison with valsartan, that is, the only study to ensure identical background renin angiotensin blocking therapy in both randomized treatment groups. The use of multi‐parametric cardiac MRI will provide high‐fidelity information about the effects of neprilysin inhibition on cardiac structure and function and, along with comprehensive biomarker profiling of patients, will provide further understanding of the mechanisms of action underlying the clinical benefits observed with sacubitril/valsartan in patients with HFrEF.

The presence of LVSD and LV dilatation are powerful predictors of the risk of developing HF following MI. Prior to reperfusion therapy, the major therapeutic breakthrough in MI was the demonstration that ACE inhibitors, given to prevent adverse LV remodelling in high‐risk patients, reduced the likelihood of developing HF and the risk of death.^5^ These benefits were seen in three seminal trials—Survival And Ventricular Enlargement (SAVE, with captopril), Acute Infarction Ramipril Efficacy, and TRAndolapril Cardiac Evaluation Study.^5^, ^11^, ^13^ In the Valsartan in Acute Myocardial Infarction Trial (VALIANT), the ARB valsartan was shown to be as effective as captopril used at the same dose as in SAVE, and the ability of ACE inhibitors and ARB to attenuate adverse LV remodelling is believed to be related to the clinical benefits of these treatments.^14^, ^28^, ^29^ Furthermore, in patients with HFrEF, the clinical benefits of ACE inhibitors, ARB, beta‐blockers, and cardiac resynchronization therapy are, in part, related to beneficial effects on adverse LV remodelling.^30^ Indeed, the effect of HFrEF treatments on LV volumes has been shown to significantly correlate with a therapy's effect on mortality in large randomized‐controlled trials.^31^

What evidence do we have for a reversal of LV remodelling with neprilysin inhibition? A series of experimental models of MI and HFrEF have reported positive effects of neprilysin inhibition on metrics of LV remodelling.^32^, ^33^ The exogenous administration of ANP has previously been reported to reduce infarct size and increase LVEF in approximately 600 patients with an acute ST‐elevation MI.^34^ Similar findings have been reported with the recombinant BNP nesiritide, and these findings suggest that the augmentation of natriuretic peptides (which is what neprilysin inhibition leads to) may have a reverse‐remodelling effect in patients with LVSD following acute MI.^35^ Several observational studies have reported favourable effects of sacubitril/valsartan on LV systolic function and volumes in patients with HFrEF (frequently with a history of prior MI), but these studies are limited in their ability to draw conclusions given the inherent limitations of such uncontrolled data.^36^, ^37^ PARADIGM‐HF did not include an echocardiographic sub‐study, and to date, two randomized‐controlled trials have reported on the effect of sacubitril/valsartan on LV remodelling in HFrEF. The Pharmacological Reduction of Functional, Ischemic Mitral Regurgitation trial compared sacubitril/valsartan with valsartan in patients with HF (defined as LVEF <50% with New York Heart Association functional classification II/III symptoms) and significant functional mitral regurgitation.^38^ Compared with valsartan, treatment with sacubitril/valsartan for 12 months significantly reduced both the degree of mitral regurgitation and LV end‐diastolic volume as measured by echocardiography. The Study of Effects of Sacubitril/Valsartan vs. Enalapril on Aortic Stiffness in Patients With Mild to Moderate HF With Reduced Ejection Fraction (EVALUATE‐HF) reported no beneficial effect of sacubitril/valsartan on the primary endpoint of central aortic stiffness.^39^ No between‐treatment difference in the pre‐specified secondary endpoint of LVEF was observed; however, sacubitril/valsartan, compared with enalapril, did improve the other pre‐specified secondary endpoints of LV and left atrial volumes after 12 weeks of follow‐up. The use of cardiac MRI imaging in our trial differentiates it to those reported earlier. Cardiac MRI is the gold‐standard method for measuring LV volumes with a superior spatial resolution and reproducibility than echocardiography. Furthermore, cardiac MRI has the additional feature of tissue characterization, allowing quantification of myocardial scar and extracellular volume fraction, which is of interest in this population given the potential anti‐fibrotic effects of sacubitril/valsartan.

There are also few data demonstrating the neurohumoral effects of neprilysin inhibition potentially underlying the clinical benefits of sacubitril/valsartan in patients with HFrEF. The substrates for neprilysin are ubiquitous and include the natriuretic peptides, adrenomedullin, endothelin, angiotensin II, substance P, bradykinin, vasoactive intestinal peptide, calcitonin gene‐related peptide, and glucagon‐like peptide‐1, among others. Following on from PARADIGM‐HF, subsequent analyses have reported only modest increases in BNP in contrast to ANP, a finding that is perhaps not unsurprising given the greater affinity neprilysin has for ANP relative to BNP.^40^, ^41^ Biomarkers of pro‐fibrotic processes (a key factor in the progression of adverse LV remodelling) have been reported to be reduced with sacubitril/valsartan compared with enalapril^42^; it is not clear, however, whether these findings reflect a direct effect of neprilysin inhibition on pro‐fibrotic signalling or are simply an indirect effect of the reduction in LV wall stress and myocardial injury as evidenced by the reductions in the N‐terminal pro‐B‐type natriuretic peptide and troponin seen with sacubitril/valsartan.^43^ It therefore remains to be seen which of the substrates for neprilysin (or which combinations) play a key role in the mechanism of action of neprilysin inhibition. Many of the peptides mentioned earlier are unable to be measured in large, multi‐centre, multi‐national randomized‐controlled trials due to their instability and difficulties in rapid measurement, as well as the need for special assays for some. The design of the present trial overcomes these hurdles, and we hope to provide novel information on the effect of neprilysin inhibition on these biomarkers and the correlation with any remodelling effect.

Several other features of the trial design merit further discussion. We recruited patients with asymptomatic LVSD identified at least 3 months after MI to ensure resolution of ‘stunning’ (reversible LVSD). This will distinguish our study from the Prospective ARNI vs. ACE Inhibitor Trial to DetermIne Superiority in Reducing Heart Failure Events After MI (PARADISE‐MI), which is enrolling patients up to 7 days after acute infarction and does not require all patients to have LVSD.^44^ A further distinguishing feature is the choice of comparator agent; unlike PARADIGM‐HF and PARADISE‐MI that used an ACE inhibitor (enalapril and ramipril, respectively), in the present trial, the use of valsartan at the dose shown to be as efficacious as captopril (target dose 50 mg three times daily) in the VALIANT will allow us to precisely define the effects of neprilysin inhibition *per se* without the uncertainty about comparing renin angiotensin system blockade with an ACE inhibitor, compared with an ARB. Our choice of primary endpoint, LV end‐systolic volume, has been shown to be a major determinant of survival after MI, and improvements in LV end‐systolic volume are associated with improved outcomes in high‐risk patients following MI.^28^, ^29^, ^45^, ^46^ We did not consider it ethical to carry out a trial like the one we are conducting in patients with symptomatic HFrEF as sacubitril/valsartan has already been shown to be definitively superior to RAAS blockade alone in those patients.^20^

## Conclusions

Despite advances in the management of acute MI, patients remain at substantial risk of developing HF as a result of residual LVSD. Inhibition of neprilysin activity in combination with RAAS blockade, using sacubitril/valsartan, has been demonstrated to improve outcomes in patients with established symptomatic HFrEF. However, the mechanisms of action underlying these clinical benefits remain unclear. They may, in part, be due to a reverse‐remodelling effect of neprilysin inhibition and increased levels of the vasoactive substrates of the enzyme, including the natriuretic peptides, among others. Results from this trial will provide comprehensive information regarding the effects of neprilysin inhibition on LV remodelling and the neurohumoral actions of sacubitril/valsartan in patients at high risk of HF following MI.

## Conflict of interest

K.F.D. reports personal fees from Eli Lilly, outside the submitted work. P.F. reports personal fees from Novartis for lectures and scientific advice, outside the submitted work. A.M. and B.S. report grants from the British Heart Foundation during the conduct of the study. P.W. reports receiving grants from Roche Diagnostics, Astrazeneca, and Boehringer Ingelheim outside the submitted work. P.S.J. reports personal fees from Novartis and payments made to his employer (the University of Glasgow) for work on the PARADIGM‐HF and PARAGON‐HF trials during the conduct of the study; personal fees from Astrazeneca; and grants from Boehringer Ingelheim, outside the submitted work. M.C.P. reports receiving grants and personal fees from Novartis lecture fees during the conduct of the study and personal fees from Novo Nordisk, AstraZeneca, Eli Lilly NAPP Pharmaceuticals, Takeda Pharmaceutical, Alnylam, Bayer, Resverlogix, and Cardiorentis and grants and personal fees from Boehringer Ingelheim outside the submitted work. J.J.V.M.'s employer employer, Glasgow University, has been paid by Novartis (who manufacture sacubitril/valsartan) for his time spent as committee member for the trials listed (using sacubitril/valsartan), meetings related to these trials, and other activities related to sacubitril/valsartan, for example, lectures, advisory boards, and other meetings. Novartis has also paid his travel and accommodation for some of these meetings. These payments were made through a Consultancy with Glasgow University, and he has not received personal payments in relation to these trials/this drug. The trials include PARADIGM‐HF: co‐PI; PARAGON‐HF: co‐PI; and PERSPECTIVE, PARADISE‐MI, and UK HARP III Trial: executive/steering committees. No other conflicts of interest were declared.

## Funding

This trial was funded by the British Heart Foundation (PG/17/23/32850), and trial medication along with funding for trial drug packaging, labelling, distribution, storage and destruction was supplied by Novartis Pharmaceuticals UK Limited who had no input to the design. J.J.V.M. and M.C.P. are supported by a British Heart Foundation Centre of Research Excellence Grant (RE/18/6/34217).

## Supporting information


**Data S1.** Cardiac MRI protocol.
**Table S1.** Total daily doses of commonly used ACE inhibitors or ARBs corresponding to ramipril 2.5mg twice daily (dose level 2 of study drug).
**Table S2.** Schedule of Assessments.
