# Supplementary material for: Rationale and methods of a randomized trial evaluating the effect of neprilysin inhibition on left ventricular remodelling
Source: ESC Heart Fail. 2020 Dec 10;8(1):129–38. doi: 10.1002/ehf2.13137 (PMC7835504; doi:10.1002/ehf2.13137)
Supplement: Supplementary file 1 — Data S1. Cardiac MRI protocol. Table S1. Total daily doses of commonly used ACE inhibitors or ARBs corresponding to ramipril 2.5mg twice daily (dose level 2 of study drug). Table S2. Schedule of Assessments. [file EHF2-8-129-s001.docx]

# **Supplementary material**

**Cardiac MRI protocol**

Cardiac MRI at baseline prior to randomisation and at 12-months following randomisation was performed on a single 3-Tesla Siemens MAGNETOM Prisma scanner at the Queen Elizabeth University Hospital Glasgow Imaging Centre of Excellence. Images were obtained with a phased-array chest coil, during breath-hold, and gated to the electrocardiogram.

A steady-state free-precession (SSFP) sequence was used to acquire long-axis (2, 3, and 4-chamber) images and a short-axis cine stack of the LV from the mitral valve plane base to the LV apex, consisting of 7mm thick slices with a 3mm interslice gap. T1 mapping images were created using a modified look-locker inversion recovery (MOLLI) sequence in the short axis view of the left ventricle at the base, mid, and apical ventricle. T1 mapping sequences were performed pre- and post-gadolinium administration. T2-mapping short axis images (base, mid and apical) were created using a fast low-angle shot (FLASH) sequence pre-gadolinium administration. Myocardial tissue tagging sequences were used to acquire 3 short axis (base, mid and apex) and three long axis (2, 3, and 4-chamber) views.

Late gadolinium enhancement images (3 long axis views, and short axis slices at the base, mid and apex) were acquired 10-15 minutes after intravenous injection of 0.15 mmol/kg of gadolinium diethyltriaminepenta-acetic acid (Gd-DTPA, Magnevist, Bayer Healthcare using segmented phase-sensitive inversion recovery turbo fast low-angle shot.

For the purposes of the measurement of the primary and secondary MRI endpoints, measurements were performed offline using the commercially available software package (Circle CVI42, Circle Cardiovascular Imaging, Canada) using standard techniques according to the Society for Cardiovascular Magnetic Resonance and European Society of Cardiovascular Imaging guidelines for reporting cardiovascular magnetic resonance examinations.^1,2^ SSFP short axis cine images were used to calculate ventricular volumes. Specifically, ventricular volumes were calculated by manually tracing the endocardial border (excluding papillary muscles and trabeculations) in end-systole and end-diastole. The basal left ventricular slice was defined as the most basal slice with >50% myocardium present. Left ventricular outflow tract volume was included in volumetric analysis. End-diastole was defined as the frame in which the blood pool of the mid ventricular slice was at its largest, and end-systole defined as the frame in which the blood pool of the mid ventricular slice was at its smallest. Values for both volumes were indexed by body surface area (BSA) calculated using the Mosteller formula, measured at the time of the scan. Left ventricular mass was calculated as the total difference between the inner and outer circumferences of the left ventricular myocardium in end-diastole, multiplied by the myocardial density (1.05g/cm^3^), indexed to BSA. Left atrial volume was calculated by manually tracing the left atrium endocardial volume in end-systole. This was performed in both the 2-chamber (vertical long axis view) and 4-chamber (horizontal long axis view). A left atrial biplane volume was then calculated using the biplane area-length method. T1 and T2 areas of interest were drawn using the afore mentioned software package. Regions of interest were drawn in myocardium remote to the area of infarction (defined as myocardium 180 degrees from area infarct site) and LV blood pool. Extracellular volume (ECV) was calculated as a ratio of corresponding T1 values measured pre- and post- contrast in each of the regions of interest. ECV was calculated using ECV = (1-HCT) × λ, where Lambda (λ)= ΔR1 myocardium /ΔR1 blood, ΔR1= R1 post-contrast- R1 pre-contrast and R1=1/T1. Haematocrit (HCT) was measured at the time of scanning. Infarct size, measured in mass and as a percentage of myocardium, was calculated by manually drawing around the epicardial and endocardial border of the late enhancement short axis images, then drawing an area of interest in normal myocardium (180 degrees from area of infarction). An auto-threshold of 5 standard deviations from this normal myocardium was used to identify areas of late enhancement. Left ventricular global function index (LVGFI) was calculated using the formula^3^:

$$LVGFI=\frac{LVEDV-LVESV}{\frac{LVEDV+LVESV}{2} +\frac{LVmass}{1.05}}\times100\%$$

Myocardial strain (global longitudinal, radial, and circumferential) was measured using feature tracking from short and long axis SSFP cine images, and also using myocardial tissue tagging sequences.

**References:**

1: Hundley WG, Bluemke D, Bogaert JG, *et al.* Society for Cardiovascular Magnetic Resonance guidelines for reporting cardiovascular magnetic resonance examinations. *J Cardiovasc Magn Reson* 2009; **11**: 5.

2: Petersen SE, Aung N, Sanghvi MM, *et al.* Reference ranges for cardiac structure and function using cardiovascular magnetic resonance (CMR) in Caucasians from the UK Biobank population cohort. *J Cardiovasc Magn Reson* 2017; **19**: 18.

3: Mewton N ,Opdahl A, Choi EY et al. Left ventricular global function index by magnetic resonance imaging--a novel marker for assessment of cardiac performance for the prediction of cardiovascular events: the multi-ethnic study of atherosclerosis. Hypertension 2013;61(4):770-8.

## **Supplement Table 1: Total daily doses of commonly used ACE inhibitors or ARBs corresponding to ramipril 2.5mg twice daily (dose level 2 of study drug)**

| **ACE inhibitor** | **Dose** | **ARB** | **Dose** |
| --- | --- | --- | --- |
| Captopril | 100mg | Candesartan | 16mg |
| Enalapril | 10mg | Irbesartan | 150mg |
| Lisinopril | 10mg | Losartan | 50mg |
| Perindopril | 4mg | Valsartan | 160mg |
| Trandolapril | 2mg |  |  |

##

## **Supplement Table 2: Schedule of Assessments**

| **Study Procedure** | **Screening**  **(>3months post myocardial infarction)** | **Visit 1 -**  **Randomisation (Week 0 [<3 months following screening])** | **Visit 2**  **(Week 1 ± 3 days)** | **Visit 3**  **(Week 2± 3 days)** | **Visit 4**  **(Week 4± 3 days)** | **Visit 5**  **(Week 5± 3 days)** | **Visit 6**  **(Week 14 ± 7 days)** | **Visit 7**  **(Week 26 ± 7 days)** | **Visit 8**  **(Week 39 ± 7 days)** | **Visit 9**  **(Week 52 ± 3 months)** |
| --- | --- | --- | --- | --- | --- | --- | --- | --- | --- | --- |
| Review Inclusion/Exclusion Criteria | X | X |  |  |  |  |  |  |  |  |
| Echocardiogram | X |  |  |  |  |  |  |  |  |  |
| Obtain informed consent | X | X |  |  |  |  |  |  |  |  |
| Cardiac MRI |  | X |  |  |  |  |  |  |  | X |
| Physical examination |  | X | X |  | X |  |  | X |  | X |
| Medical history | X | X |  |  |  |  |  |  |  |  |
| Concomitant medications | X | X | X | X | X | X | X | X | X | X |
| Vital Signs (Blood pressure/Heart rate) | X | X | X | X | X | X | X | X | X | X |
| 12 Lead ECG |  | X |  |  |  |  |  |  |  |  |
| Spot urine collection |  | X |  |  |  |  |  | X |  | X |
| Venepuncture (Urea and Electrolytes/FBC and LFTs) | X^&^ | X | X | X | X | X | X | X | X | X |
| Venepuncture (Biochemical/hormonal/  biomarker analysis) |  | X |  |  |  |  |  | X |  | X |
| Pregnancy testing in WoCBP |  | X |  |  | X |  | X | X | X | X |
| Patient global assessment questionnaire |  |  |  |  |  |  |  |  |  | X |
| IMP Dispensing* |  | X |  |  | X |  | X | X |  |  |
| Up-titrate IMP# |  |  |  |  | X |  |  |  |  |  |
| IMP Administration+ |  | X | X | X | X | X | X | X | X | X |
| Adverse event reporting |  |  | X | X | X | X | X | X | X | X |
| Study completion |  |  |  |  |  |  |  |  |  | X |

^*^ Study drug will be introduced at equivalent dose to existing ACE-i/ARB treatment (Dose level 2 or 3) at investigator’s discretion

^#^Up-titration not required if patient already on dose level 3

^&^If not done in the preceding 6 months

**Patient global assessment of change questionnaire**
